# Supplementary material for: Multi-level interaction between HIF and AHR transcriptional pathways in kidney carcinoma
Source: Life Sci Alliance. 2023 Feb 1;6(4):e202201756. doi: 10.26508/lsa.202201756 (PMC9896012; doi:10.26508/lsa.202201756)
Supplement: Supplementary file 1 [file LSA-2022-01756_TableS1.docx]

TABLE S1 List of primers for RT-qPCR analysis

|  | Gene | Sequence |
| --- | --- | --- |
| AHR target genes | *CYP1B1* | Sense 5’- GAGAACGTACCGGCCACTATC-3’  Antisense 5’- CGACCTGATCCAATTCTGCCT-3’ |
|  | *ALDH1A3* | Sense 5’- TGTAACCCTTCAACTCGGGA -3’  Antisense 5’- CCTCTGGAAGGCAACCTGT -3’ |
|  | *CYP27A1* | Sense 5’- GACCAGCACGACCTGACCTA -3’  Antisense 5’- TCCGTATAGAGCGCTGCTTC -3’ |
|  | *BMF* | Sense 5’- AAGGTGTCATGCTGCCTTGT -3’  Antisense 5’- CAAGACTGCTGGGAAACTGG -3’ |
|  | *ASB2* | Sense 5’- CCGGACATCTCCAACAAATC -3’  Antisense 5’- TGGTGTCTGCATTGTGCTG -3’ |
| HIF target genes | *NDRG1* | Sense 5′- TAACGTGGAAGTGGTCCACAC -3′  Antisense 5′- ATTGGTCGCTCAATCTCCAG -3 |
|  | *PFKFB4* | Sense 5′- AATTATCCACTGGAGTTCGCC -3′  Antisense 5′- AGCTCCATGATGACAGGCTC -3′ |
|  | *INSIG2* | Sense 5′- ATTGGTCGACAACTGGCAAT -3′  Antisense 5′- TTTTGCCTTCTTCATTCCTGAT -3′ |
|  | *ANKRD37* | Sense 5′- CGCTGACCTCAACCAGCA -3′  Antisense 5′- AATTTGGGCATCACTGGCTA -3′ |
|  | *ARL10* | Sense 5′- CCTGACCTGCCTGTCGTC -3′  Antisense 5′- AAAACCTCCCGCTGGTTATC -3′ |
|  | *HPRT1* | Sense 5′- CTGAGGATTTGGAAAGGGTGT-3′  Antisense 5′- CATCTCGAGCAAGACGTTCA-3′ |
